# Differential influence of 1,8-Cineol on distinct hypoxia-related immune alterations in human monocytes

**DOI:** 10.1038/s41598-025-97314-7

**Published:** 2025-04-09

**Authors:** Nele Jagodzinski, Anke Leichtle, Reinhard Depping, Kirstin Plötze-Martin, Samer G. Hakim, Karl-Ludwig Bruchhage, Ralph Pries

**Affiliations:** 1 Department of Otorhinolaryngology, University Hospital of Schleswig-Holstein, Campus Lübeck, Ratzeburger Allee 160, 23538 Lübeck, Germany; 2 Center for Structural and Cell Biology in Medicine, Institute of Physiology, Working Group Hypoxia, University of Lübeck, Lübeck, Germany; 3 Department of Oral and Maxillofacial Surgery and Plastic Reconstructive Head and Neck Surgery, Helios Medical Center, Schwerin, Germany; 4 Department of Maxillofacial Surgery, University of Lübeck, Lübeck, Germany

**Keywords:** Hypoxia, 1,8-Cineol, Monocytes, Cytokines, Adhesion molecules, FGF-7, Cell biology, Drug discovery

## Abstract

1,8-Cineol is a natural plant-based therapeutic agent and is commonly used to treat a broad range of acute and chronic airway inflammatory diseases. 1,8-Cineol has recently been shown to attenuate the checkpoint molecule PDL-1 in circulating monocytes in patients with chronic Otitis media (OM) and was associated with an improved clinical outcome. Hypoxia-inducible factor (HIF) is thought to play an essential role in the middle ear inflammatory process, mainly due to dysfunctions of the eustachian tube. However, the unambiguous impact of 1,8-Cineol on hypoxia-driven immune alterations of human monocytes and the related inflammatory microenvironment have not been investigated thus far. Therefore, we used the human monocytes to investigate the impact of 1,8-Cineol on the cellular hypoxia response with regards to expression levels of different adhesion molecules, chemokine receptors, and different cell stress-related proteins. Furthermore, the secretion patterns of a variety of chemokines and cytokines were evaluated. The study aimed to better understand the influence of the monoterpene 1,8-Cineol on hypoxia and normoxia-associated monocyte characteristics and related inflammatory processes, all of which are crucial for the development of various human diseases.

## Introduction

The monoterpene 1,8-Cineol is a well-studied natural plant-based active substance, which combines different anti-microbial and anti-inflammatory properties and is commonly used as the clinically approved drug Soledum^®^ to treat a broad range of acute and chronic airway inflammatory disorders^1^. Recent studies revealed a wide systemic distribution of 1,8-Cineol in the human organism, improving the individual immunologic situation e.g. via monocyte derived inflammatory cytokines^2–6^. Monocytes are a primary source of inflammatory cytokines and essential regulators of the initiation of further adaptive immune responses^7^. Additionally, 1,8-Cineol has recently been shown to attenuate the checkpoint molecule PD-L1 and adhesion molecule CX3CR1 in circulating monocytes in patients with chronic Otitis media (OM), which is a widespread inflammatory disease of the middle ear and often causes hearing impairment, difficulties in conversations and social distancing^8–10^. A lack of treatment may finally lead to otogenic complications such as mastoiditis, labyrinthitis, thrombosis of venous sinus or intracranial complications^11,12^. Recently, the anti-inflammatory plant-based drug 1,8-Cineol has been shown to improve the microbiota distribution of the middle ear, the intestinal bacterial colonization as well as the clinical course of chronic Otitis media patients^13^. Moreover, treatment of Otitis media patients with 1,8-Cineol revealed a significant reduction of inflammatory microbes in ear samples and was associated with an improved clinical outcome^13^. In the context of an OM infection, hypoxia-inducible factor (HIF) is thought to play an important role in the middle ear inflammatory process due to the inherent dysfunctions of the eustachian tube^14–16^. Increased abundances HIF-1α in middle ear effusion has also been associated with the pathology of an impaired bone conduction in Otitis media with effusion^17^. Intermittent hypoxia has been linked to an upregulation of HIF-1α, which induces an increase of PD-L1 expression on monocytes and T cells^18^, leading to a modulation of the immune response. Furthermore, hypoxia triggered monocytes revealed increased secretion levels of chemokine CXCL4, which also has been shown to be produced by umbilical cord CD34 derived plasmacytoid dendritic cells via an overproduction of mitochondrial reactive oxygen species (mtROS) as well as a stabilization of HIF-2α^19,20^. Overall, hypoxia is closely associated with various biological processes, such as bacterial infections, metabolism, cancer, inflammatory diseases, and cellular stress responses^21^. Correspondingly, studies show that an obstructive sleep apnoea syndrome (OSAS) related systemic hypoxia leads to an alteration of different immune cells such as lymphocytes, NK cells and monocytes as well as increased levels of inflammatory cytokines and checkpoint molecule PD-L1 on circulating monocytes^7,22–26^. An anti-inflammatory activity of 1,8-Cineol has been described in different inflammatory diseases by modulating various associated biosynthetic pathways^3,27–29^. Greiner and colleagues described for the first time a significant downregulation of inflammatory processes via decreased activities of transcription factor NFκB and the JNK (c-Jun N-terminal kinase)/AP-1 (activator protein-1) pathway in the human cancer cell lines U373 and HeLa in response to 1,8-Cineol^30^. Furthermore, the Egr-1 (early growth response-1) MAPK pathway regulated transcription factor, that plays an important role in the regulation of many inflammation associated genes, has been shown to be inhibited by 1,8-Cineol in human monocytes^31,32^.

However, the unambiguous impact of 1,8-Cineol on hypoxia-driven immune alterations of human monocytes and the related inflammatory microenvironment have not been investigated thus far. Therefore, we used the human monocyte leukemia cell line THP-1 (Tohoku Hospital Pediatrics-1)^33^ as a model to investigate the impact of 1,8-Cineol on the modulation of the cellular hypoxia response with regard to the expression of different adhesion molecules, chemokine receptors, checkpoint molecule PD-L1 and cell stress-related proteins. More precisely, monocytes were analyzed by flow cytometry with regard to surface expression levels of adhesion molecules CD11a (integrin-α L; LFA-1), CD11b (integrin-α M; Mac-1), CD11c (integrin-α X), CX3CR1 (CX3CL1 receptor) all of which are known to be differentially regulated in response to inflammatory condition and closely associated with the closely associated with the transendothelial migration of myeloid cells^34^. Furthermore, comprehensive screening of secretion patterns of a variety of chemokines and cytokines with regard to hypoxia and the influence of 1,8-Cineol was carried out.

For the condition of intermittent hypoxia (5% O_2_) in the presence and absence of 1,8-Cineol, THP-1 cells were incubated in a humidified hypoxia chamber followed by a recovery phase under normoxic conditions. In addition, the hypoxia mimetic cobalt chloride (CoCl_2_), which stabilizes hypoxia inducible factors (HIF) -1α and − 2α under normoxia conditions, was used as an internal control^35^.

## Results

### Hypoxia-related alteration of human monocytes

To investigate the impact of 1,8-Cineol on intermittent hypoxia driven immune alterations, THP-1 monocytes were (I) incubated in the presence of 100µM CoCl_2_ for 1 h followed by a CoCl_2_ removal (‘normoxia’ in form of fresh non-CoCl_2_-containing medium) for 1 h or (II) cells were incubated at 5% O_2_ in a hypoxia chamber for 1 h followed by an incubation at normoxia conditions for 1 h. Hypoxia conditions were induced in the presence and absence of 100µM 1,8-Cineol (CNL-1976^®^) and compared to the normoxia control. As an internal control, induction of intermittent hypoxia in response to 1 h CoCl_2_ and 5% O_2_ was confirmed via quantitative PCR of hypoxia-inducible gene Glut1 (data not shown).

**Fig. 1:**
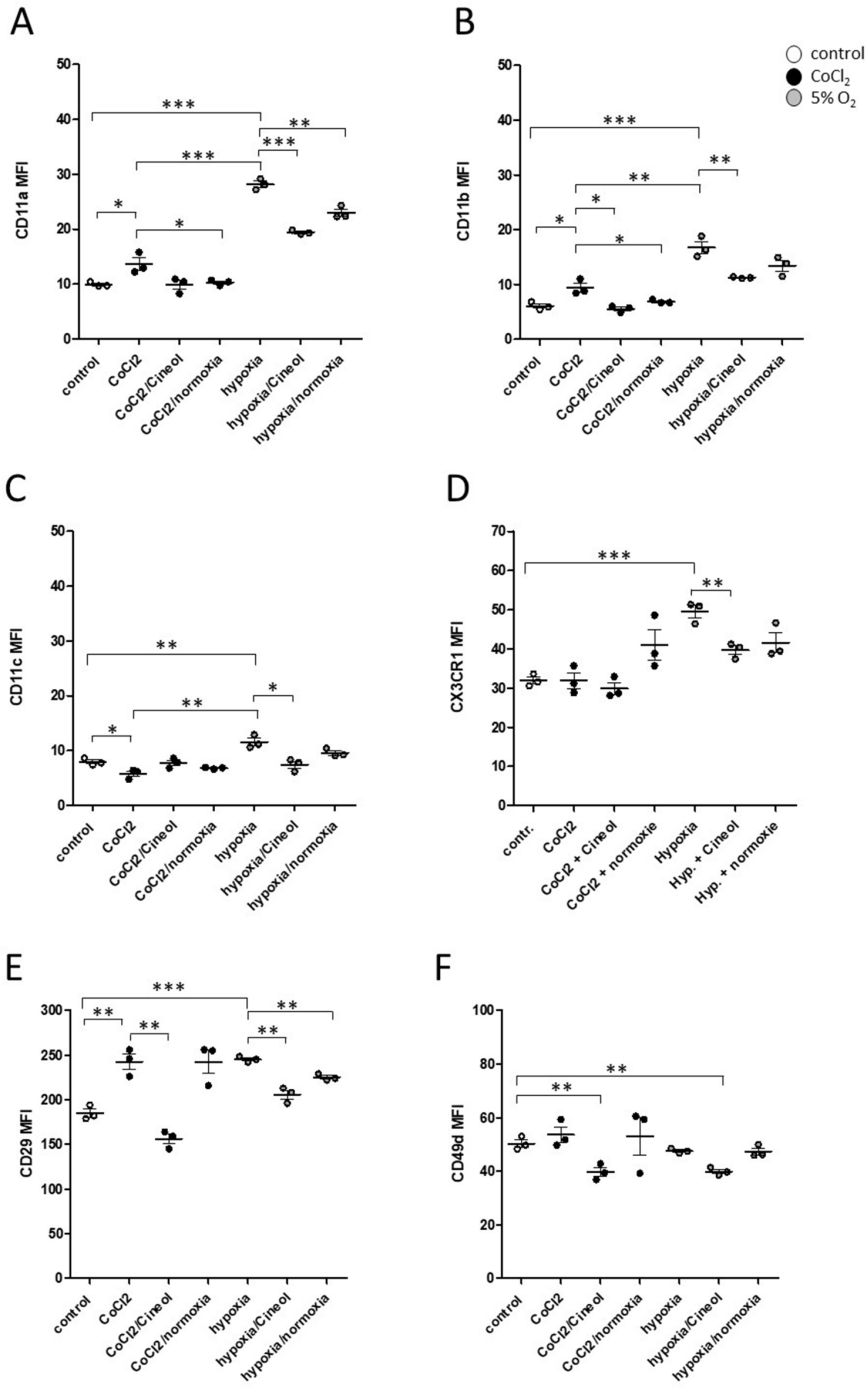
Flow cytometric analyses of monocytic adhesion molecules upon hypoxia induction. (**A/B**): Expression levels of CD11a and CD11b were significantly elevated in response to CoCl_2_ incubation and even stronger by hypoxia (5% O_2_) conditions, which could clearly be attenuated by both 1,8-Cineol and normoxia conditions compared to the control. (**C/D**) Expression levels of adhesion molecules CD11c and CX3CR1 were significantly increased in response to hypoxia (5% O_2_) conditions, whereas increased levels could not be observed in the presence of 1,8-Cineol or upon normoxia conditions. (**E**) Expression of adhesion molecule CD29 was significantly increased in response to CoCl_2_ incubation, which could clearly be inhibited by 1,8-Cineol but was not decreased upon 1 h of normixia condition. Increased CD29 levels were also observed after 1 h of hypoxia (5% O_2_) conditions, which could not be observed in the presence of 1,8-Cineol or upon normoxia conditions. (**F**) Expression levels of adhesion molecule CD49d were not induced by CoCl_2_ or hypoxia (5% O_2_), but significantly reduced in response to 1,8-Cineol. MFI: mean fluorescence intensity. *: *p* < 0.05; **: *p* < 0.01; ***: *p* < 0.001.

Expression levels of adhesion molecules and chemokine receptors CD11a (integrin-α L; LFA-1), CD11b (integrin-α M; Mac-1), CD11c (integrin-α X), CX3CR1 (CX3CL1 receptor), CD29 (integrin β-1), and CD49d (integrin β-4) were investigated. Expression levels of CD11a and CD11b were found to be significantly elevated in response to CoCl_2_ incubation and to a higher extent by hypoxia (5% O_2_) conditions, which could be attenuated by both 1,8-Cineol and normoxia conditions (Fig. 1A/B).

Expression levels of adhesion molecules CD11c and CX3CR1 were significantly increased in response to hypoxia (5% O_2_) conditions, whereas increased levels could not be observed in the presence of 1,8-Cineol or upon normoxia conditions (Fig. 1C/D). Expression of adhesion molecule CD29 was significantly increased in response to CoCl_2_ incubation, which could clearly be inhibited by 1,8-Cineol but was not decreased upon 1 h of normoxia condition. Increased CD29 levels were also observed after 1 h of hypoxia (5% O_2_) conditions, which could not be observed in the presence of 1,8-Cineol or upon subsequent normoxia conditions (Fig. 1E). In contrast, data revealed significantly decreased expression levels of adhesion molecule CD49d in response to both CoCl_2_ and hypoxia conditions in the presence of 1,8-Cineol (Fig. 1F).

**Fig. 2:**
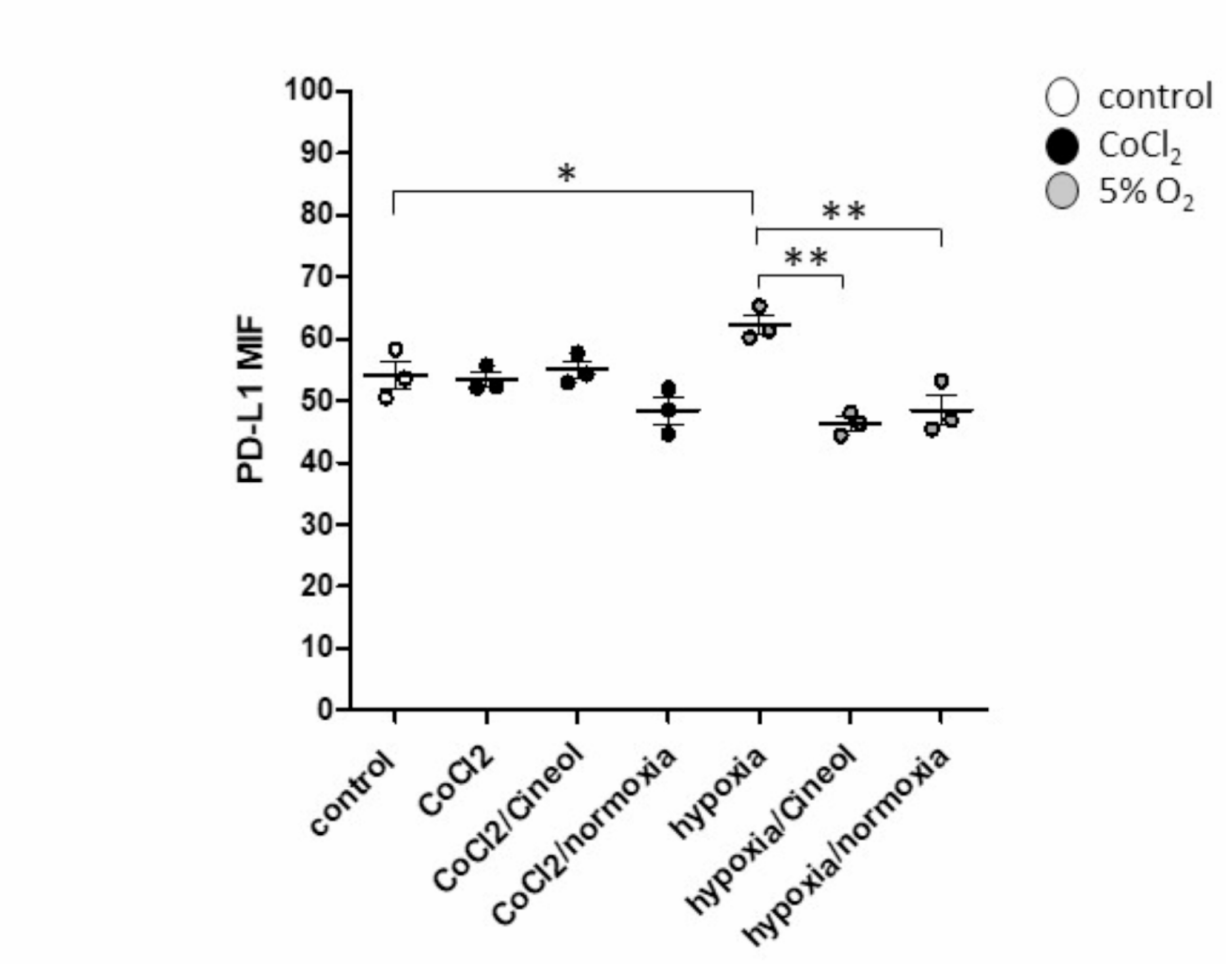
Hypoxia-related expression of checkpoint molecule PD-L1. Flow cytometric analyses revealed significantly increased PD-L1 expression levels in response to hypoxia conditions (5% O_2_) compared to the normoxic control, which were found to be significantly decreased by both 1,8-Cineol and normoxic conditions. In contrast, PD-L1 expression on THP-1 monocytes was not influenced upon 1-hour incubation with CoCl_2_. MFI: mean fluorescence intensity. *: *p* < 0.05; **: *p* < 0.01.

Next, monocytic expression patterns of checkpoint molecules PD-L1 were analyzed using flow cytometry. Data revealed significantly increased PD-L1 expression levels in response to hypoxia conditions (5% O_2_) compared to the normoxia control, which were found to be significantly decreased by both 1,8-Cineol and normoxia conditions. In contrast, PD-L1 expression on THP-1 monocytes was not influenced upon 1-hour incubation with CoCl_2_ (Fig. 2).

**Fig. 3:**
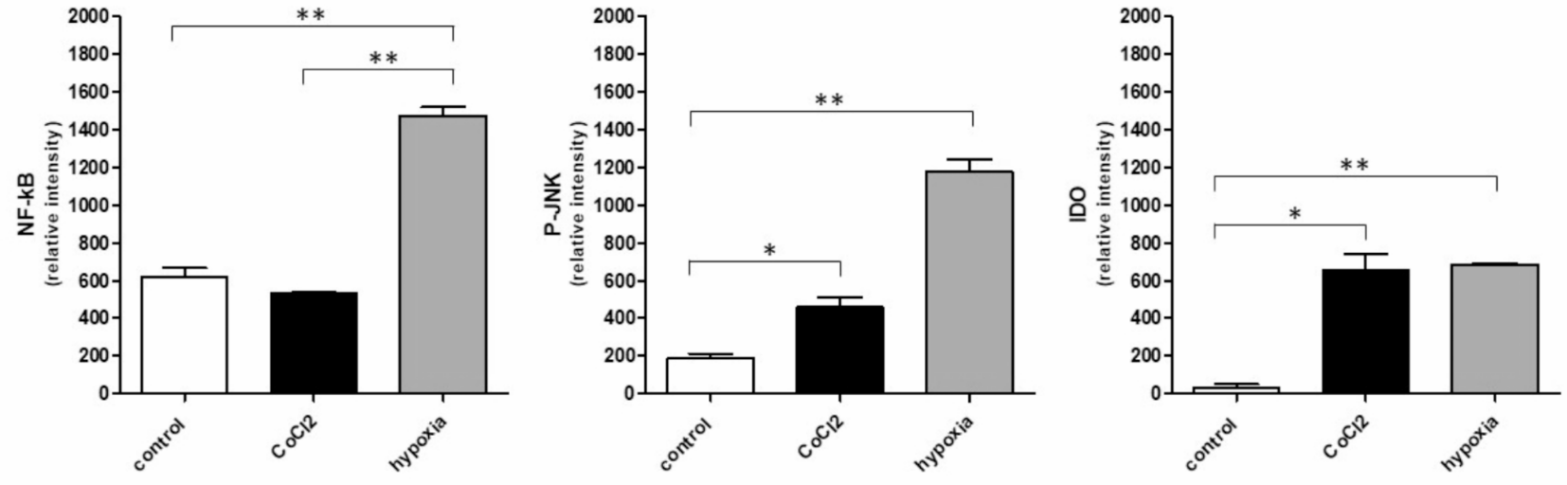
Hypoxia related cell stress response. Semiquantitative analysis by measuring the relative intensity of the dots revealed differential expression patterns of NF-kB (nuclear factor kappa B), P-JNK (c-Jun N-terminal kinase), and IDO (indoleamine 2, 3-dioxygenase) in response to CoCl_2_ and hypoxia (5% O_2_) compared to the normoxic control. *: *p* < 0.05; **: *p* < 0.01.

To further investigate potential factors that may be involved in the observed differential influence of 1,8-Cineol on hypoxia- and CoCl_2_-driven immune alterations in human monocytes, expression patterns of different cell stress-related proteins were analyzed using membrane based human proteome profiler arrays. Semiquantitative analyses of monocyte cell lysates revealed differential activation patterns of transcriptional activator proteins NF-κB (nuclear factor kappa B) and P-JNK (c-Jun N-terminal kinase), and heme dioxygenase IDO (indoleamine 2, 3-dioxygenase) in response to CoCl_2_ and hypoxia (5% O_2_) compared to the normoxia control. As can be seen in Fig. 3, the expression levels of NF-κB and P-JNK were higher in response to hypoxia (5% O_2_) compared to the control and influence of CoCl_2_, whereas CoCl_2_ and hypoxia had a similar effect on the expression of IDO (Fig. 3).

### Hypoxia-induced cytokine secretion

**Fig. 4:**
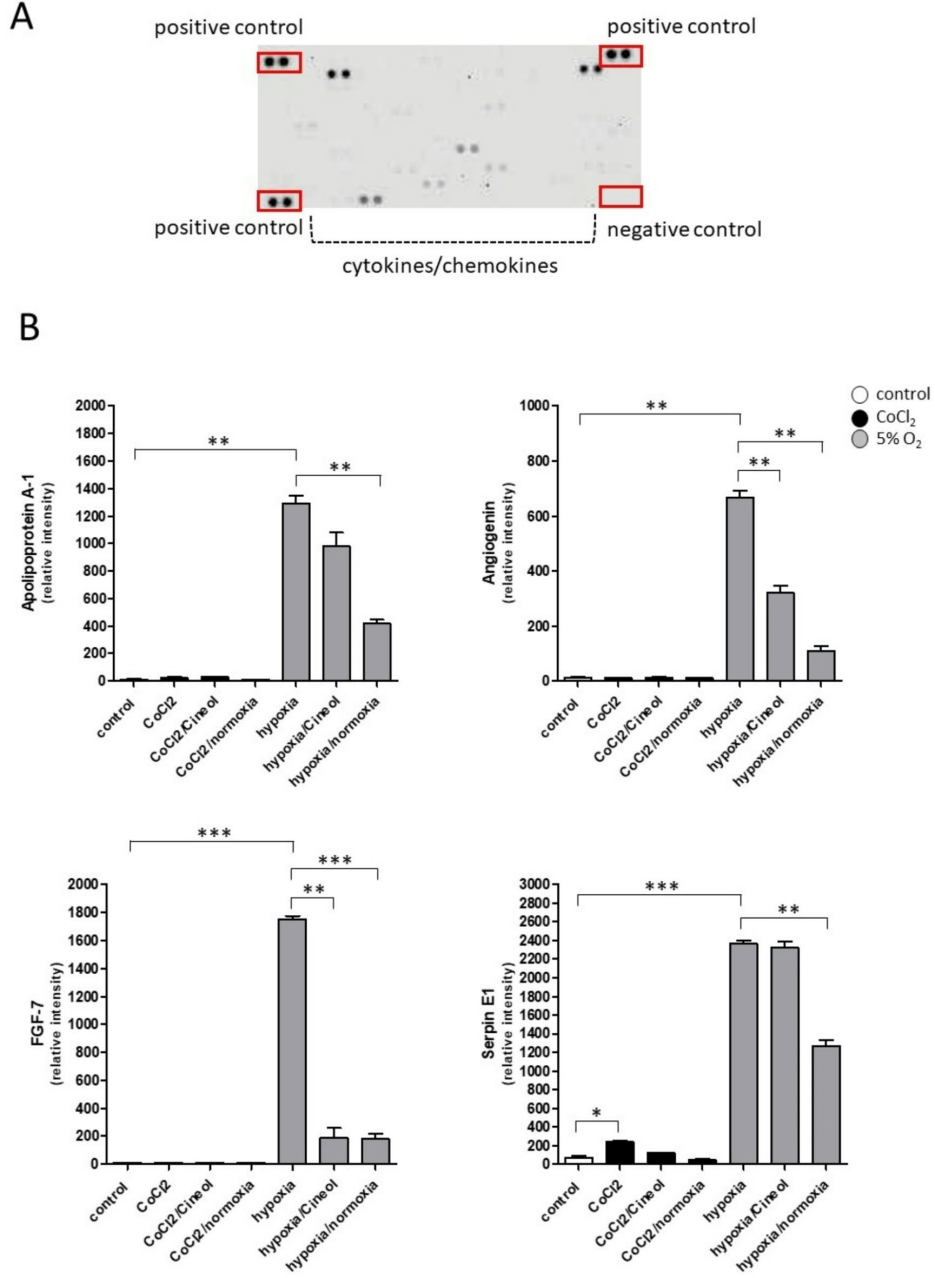
Hypoxia-associated cytokine secretion pattern. (**A**) Example image of a membrane based cytokine array of THP-1 cell culture supernatants illustrating the membrane-organisation of positive and negative controls and cytokine dots. (**B**) Semiquantitative analysis was performed by measuring the density of the dots and revealed differential expression patterns of certain cytokines (Apolipoprotein A-1; Angiogenin; Fibroblast growth factor 7 (FGF-7); Serpin E1) in response to CoCl_2_, CoCl_2_/Cineol, CoCl_2_/normoxia, hypoxia (5% O_2_), hypoxia/Cineol, hypoxia/normoxia compared to the normoxia control. *: *p* < 0.05; **: *p* < 0.01; ***: *p* < 0.001.

The identified proteins NF-κB (nuclear factor kappa B), P-JNK (c-Jun N-terminal kinase), and IDO (indoleamine 2, 3-dioxygenase) are known to be activated by environmental stress and inflammatory cytokines. Therefore, secretion patterns of 105 different cytokines and chemokines were analyzed using membrane-based human cytokine arrays to further improve the understanding of the influence of 1,8-Cineol on hypoxia driven immune alterations in human monocytes. Data revealed differential secretion patterns of cytokines Apolipoprotein A-1, Angiogenin, Fibroblast growth factor 7 (FGF-7), and Serpin E1, all of which were highly increased in response to hypoxia (5% O_2_) and strongly decreased through a subsequent normoxia incubation. Furthermore, Hypoxia (5% O_2_) driven secretion of fibroblast growth factor 7 (FGF-7) could be entirely prevented by 1,8-Cineol, which in turn had no effect on hypoxia-driven Serpin E1 secretion (Fig. 4).

Cytokines Apolipoprotein A-1 and Angiogenin revealed strongly reduced secretion levels upon subsequent normoxia conditions after hypoxia 5% O_2_ and slightly reduced levels in the presence of 1,8-Cineol. Among these cytokines only Serpin E1 was found to be slightly increased in response to CoCl_2_ incubation and decreased upon CoCl_2_ removal (Fig. 4).

**Fig. 5:**
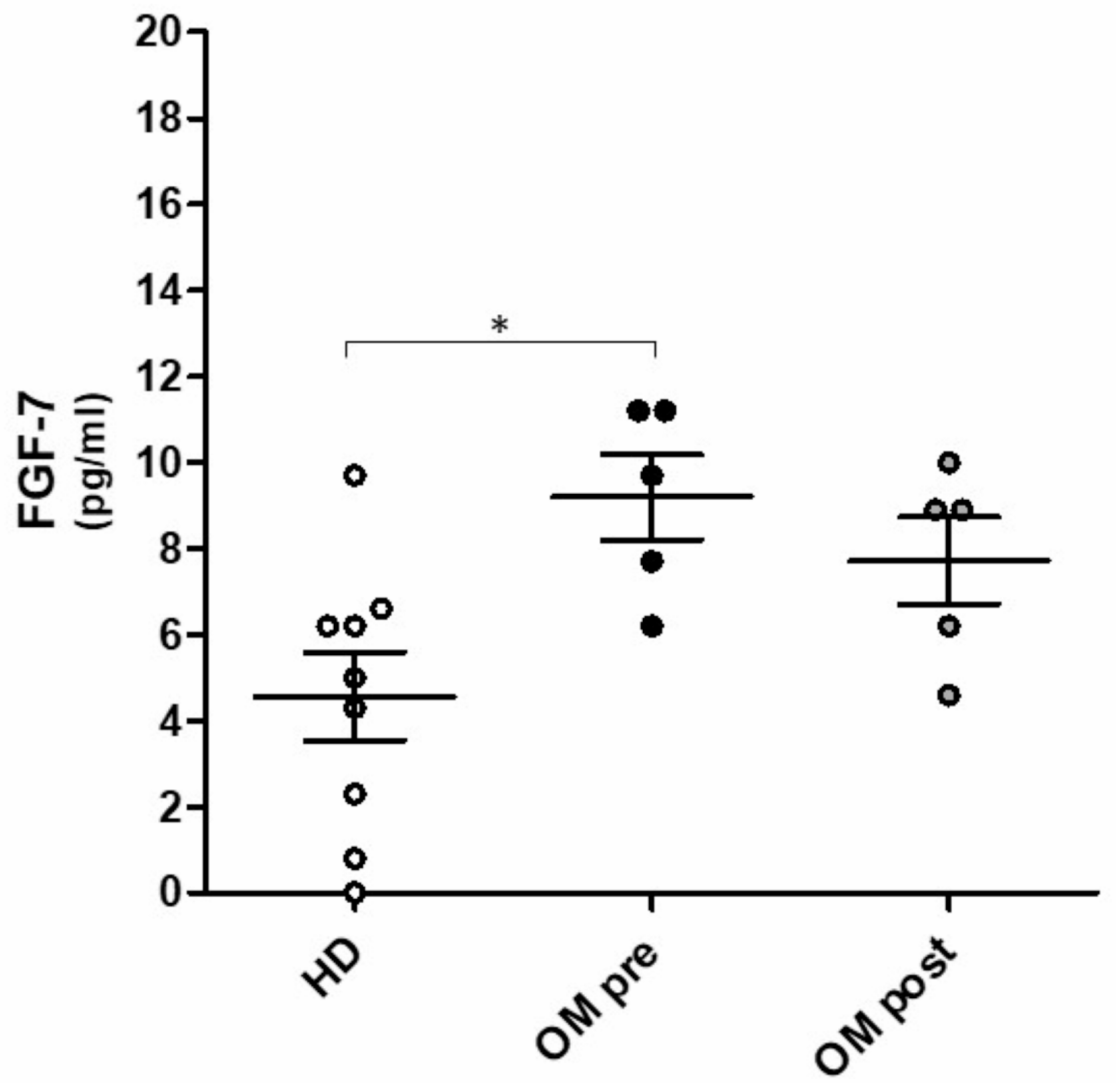
ELISA measurements of plasma FGF-7 (Fibroblast growth factor 7). (**A**) Data revealed significantly increased FGF-7 plasma levels (pg/ml) in Otitis media patients (*n* = 5) before 1,8-Cineol treatment (OM pre) compared to healthy donors (HD; *n* = 9) and slightly reduced levels after 1,8-Cineol treatment (OM post). *: *p* < 0.05.

Of note, the family of fibroblast growth factors (FGFs) comprises 22 members and is critically involved in various biological functions such as cell growth, wound healing as well as hyperproliferative epithelial diseases. Therefore, some initial plasma samples of Otitis media patients were examined and revealed significantly increased levels of fibroblast growth factor 7 (FGF-7) in comparison to healthy donors as well as slightly reduced levels upon 1,8-Cineol treatment (Fig. 5).

## Discussion

### Impact of 1,8-Cineol on hypoxia-driven cellular characteristics

The aim of this study was to investigate the influence of anti-inflammatory monoterpene 1,8-Cineol (CNL-1976^®^) on hypoxia-driven immune alterations of human monocytes. It is well known that hypoxia conditions activate various signaling pathways in mammalian cells, mainly via hypoxia-inducible factor 1α (HIF1α)^36,37^. The transcription factor HIF-1α is expressed in many different immune cells such as dendritic cells (DCs), neutrophils, and macrophages. It enables the cellular response to hypoxia conditions by regulating different processes such as proliferation and inflammation^38,39^. In this context, HIF-related transcriptional activation is closely associated with cardiovascular, metabolic, inflammatory, and infection-related diseases^40–42^, whereas HIF-1α hydroxylation under normoxia conditions inhibits the recruitment of involved transcriptional coactivators^43^.

Our data revealed elevated levels of leukocyte adhesion molecules CD11a and CD11b in response to CoCl_2_ incubation and hypoxia (5% O_2_) conditions, which was attenuated by 1,8-Cineol and normoxia conditions. CD11a and CD11b are associated with the adhesion of inflammatory monocytes to endothelial cells in patients with coronary artery diseases^44^. Furthermore, expression levels of adhesion molecules CD11c and CX3CR1 significantly increased in response to hypoxia conditions, whilst an increase could not be shown under the presence of 1,8-Cineol or in response to normoxia conditions. It has been shown, that CD11c expressing innate immune cells are closely associated with the inflammatory situation in adipose tissues^45^. In contrast, the adhesion molecule CX3CR1 is essential for monocyte crawling along the blood vessels and is associated with atherosclerosis and vascular inflammation^46–48^. Hypoxia conditions also triggered increased CD29 expression, which is another essential regulator of cell-adhesion between leukocytes and endothelial cells^49^. This increase could also be prevented by 1,8-Cineol and normoxia conditions. These data indicate an anti-inflammatory impact of 1,8-Cineol on the regulation of hypoxia-driven inflammatory processes. Furthermore, hypoxia conditions resulted in a significant upregulation of checkpoint molecule PD-L1 on THP-1 monocytes and significantly decreased by 1,8-Cineol and normoxia conditions. These data are in line with observations that transcriptional activator protein NFκB regulates PD-L1 in non-small cell lung carcinoma during EMT signaling^50^ and that 1,8-Cineol leads to a reduced nuclear translocation of NFκB and a decreased transcription of target genes in lipopolysaccharide (LPS) stimulated cells^30^. In contrast, PD-L1 expression on THP-1 monocytes was not influenced upon 1-hour incubation with CoCl_2_. Previous publications indicated NF-κB as a key component within hypoxia-driven HIF1α mediated inflammatory signaling pathways, whereas the activation mechanism of NF-κB upon hypoxia conditions is still not completely understood^51,52^. According to our observations, it has been discovered, that CoCl_2_ attenuates oxidative stress and inflammation through NF-κB inhibition in human renal proximal tubular epithelial cells^53^.

Moreover, our data revealed increased activation levels of cell stress-related proteins IDO (indoleamine 2, 3-dioxygenase) and P-JNK (phosphorylated c-Jun N-terminal kinase) in response to both CoCl_2_ and hypoxia (5% O_2_). Indoleamine 2,3-dioxygenase (IDO) is a heme-containing intracellular dioxygenase catalyzing the degradation of the essential amino acid L-tryptophan to N-formyl-kynurenine. IDO is widely expressed in dendritic cells, macrophages, microglia, eosinophils, fibroblasts, endothelial cells, and most tumor cells. It has been shown that hypoxia enhances IDO production, which induces immunosuppression and mediates T-cell tolerance by dendritic cells^54,55^. The influence of hypoxia on distinct basic inflammatory biosynthetic pathways underlines the potential impact of local hypoxia driven inflammations on the systemic immunological situation. Otitis media with effusion has been described as an initial manifestation of granulomatosis with polyangiitis, which is an autoimmune disease affecting vessels of the respiratory tract, kidneys, and other organs and thus underlines the systemic influence of local hypoxia related inflammation^56^. Moreover, an association of OM and rheumatoid arthritis has been described^57^.

Similarly, it has recently been shown that chronic intermittent hypoxia-induced oxidative stress activates P-JNK to mediate insulin resistance and cell apoptosis in the pancreas^58^ which respectively regulates the synthesis of many inflammatory cytokines^59^.

### Impact of 1,8-Cineol on hypoxia-driven cytokine secretion

Hypoxia-associated pro-inflammatory cytokines are well known to contribute to immune disturbances and the development of different diseases^60–62^. Our data revealed differential secretion patterns of cytokines Apolipoprotein A-1 (ApoA1), Angiogenin, Fibroblast growth factor 7 (FGF-7), and Serpin E1, all of which were increased in response to hypoxia and highly decreased by a subsequent normoxia incubation. Apo1 has been suggested as a promising novel biomarker for the diagnosis of coronary artery disease^63^. It has also been shown that ApoA1 is associated with pathological angiogenesis in hypoxia-induced human retinal vascular endothelial cells by inhibiting ERK1/2 signaling^64^. In a recent study, hypoxia-mimicking agent CoCl_2_ has been shown to induce the accumulation of intracellular and membrane protein ApoA-1 in THP-1 macrophages. In contrast, the three major MAP-kinase cascades (ERK1/2, JNK1/2/3, and p38) and the NF-κB transcription factor are involved in the hypoxia-induced *ApoA-1* gene expression^65^.

In alignment with our data, it has been shown that hypoxia-induced reactive oxygen species (ROS) in breast cancer cells, selectively regulate hypoxia-induced increases in N-cadherin and Serpin E1, two proteins involved in cell adhesion^66^. Besides the regulatory impact of 1,8-Cineol on the translocation and activity of different MAPK dependent transcriptional activators, it also suppresses the activation of the NOD-like receptor pyrin domain-containing 3 (NLRP3) mediated formation of the inflammasome^67^. The NLRP3 inflammasome has also been associated with human middle ear cholesteatoma and chronic otitis media^68^.

Although Cobalt chloride (CoCl_2_) is a well-known and feasible hypoxia mimetic mediator that induces hypoxia-like responses, various differential effects of CoCl_2_ induced hypoxia conditions have been reported. For instance, it has been shown that simulating hypoxia by CoCl_2_ can effectively increase hypoxia-associated genes, specially HIF-1α and GLUT-1, but did not affect HIF-2α gene expression^69^. Furthermore, CoCl_2_ revealed divergent effects with regard to cell migration and glycolysis compared to physical hypoxia^70^. From this point of view, the present study underlines these differential effects of real hypoxia condition and the hypoxia mimetic CoCl_2_, which should be kept in mind in future in vitro investigations.

Moreover, data revealed that hypoxia-driven secretion of fibroblast growth factor 7 (FGF-7) could be prevented by 1,8-Cineol in vitro. Of note, the family of fibroblast growth factors (FGFs) consists of 22 members and is critically involved in various biological functions. For instance, this includes cell growth, wound healing as well as hyperproliferative epithelial diseases, as a consequence of the activation of dermal fibroblasts by the inflammatory microenvironment. In this context, cholesteatoma-associated fibroblasts have been shown to modulate epithelial growth and differentiation through FGF-7 secretion^71^.

Our initial investigations revealed significantly increased levels of fibroblast growth factor 7 (FGF-7) in plasma samples of Otitis media patients compared to healthy donors and slightly reduced levels upon 1,8-Cineol treatment. It has recently been shown, that HIF1α also contributes to the proliferation of cholesteatoma keratinocytes, which is a hyperproliferative, pseudoneoplastic lesion of the middle ear characterized by aggressive growth and bone destruction^72^.

Further comprehensive studies on larger patient cohort have to be carried out to elucidate the potential role of plasma FGF-7 in regulating OM associated inflammation and cholesteatoma development with regards to 1,8-Cineol treatment. An acknowledged limitation of the study is the lack of measurements of the transcriptional activation and protein accumulation of HIF proteins. Future investigations on larger patient cohorts are needed to corroborate our findings and to further correlate these data with the regulation of HIF transcriptional activators and individual clinical parameters.

In summary, our study provides novel insights into the complex regulation of hypoxia-driven inflammation and the potential differential effects of monoterpene 1,8-Cineol on associated systemic balances of inflammatory mediators and human monocytes. It was shown that the effect of 1,8-Cineol on hypoxia-related processes was often similar to the effect of subsequent normoxia.

For instance, 1,8-Cineol had a significant impact on the suppression of hypoxia related upregulation of certain adhesion molecules (such as CD11a, CD11b, CD11c, CX3CR1) as well as the checkpoint molecule PD-L1. Regarding the impact on cytokine secretion, normoxia had a stronger effect on the downregulation of secretion patterns than 1,8-Cineol for the most part, nonetheless a reduced effect by 1,8-Cineol was still observed.

In general, our data indicates that there is an anti-inflammatory impact of 1,8-Cineol on the regulation of hypoxia driven inflammatory processes.

## Materials and methods

### Ethics statement and blood collection

Patients with Otitis media were examined at the Department of Otorhinolaryngology, University Hospital Schleswig-Holstein, Campus Luebeck, after their written informed consent. The study was approved by the ethics committee of the University of Luebeck (approval number 21–183) and performed according to the ethical principles of the WMA Declaration of Helsinki.

Blood samples were obtained from healthy donors (*n* = 9; 5 female/4 male) and patients with chronic Otitis media (*n* = 5; 2 female/3 male) before and after 14 days of 1,8-Cineol administration. The median age of the patients with COM was 53 years and all patients initially underwent antibiotic therapy and middle ear surgery and developed microbial resistances against many or all available classical antibiotics. Often, there were no therapeutic options left and therefore we treated them with 1,8-Cineol as part of the routine treatment at our institution.

1,8-Cineol (CNL-1976) was used in terms of the clinically approved drug Soledum^®^ Kapseln forte (capsules) (Cassella-med GmbH & Co. KG, Cologne, Germany). For therapeutic use patients have been prescribed Soledum capsules forte (3 × 200 mg Cineol/day) over 14 days for oral administration. They did not obtain any local or systemic antibiotic treatments during this time period.

### THP-1 cells and culture conditions

For cell culture experiments the non-adherent monocyte cell line THP-1 (Tohoku Hospital Pediatrics-1) was used. Cell culture experiments were performed in triplicate and we used RPMI 1640 medium supplemented with 10% heat inactivated fetal bovine serum (FBS), 1% sodium pyruvate and 1% streptomycin/penicillin at 37 °C and 5% CO_2_ under a humidified atmosphere. Cells were subcultured every 3 days when they reached a maximum density of 1 × 10^6^ cells/ml. For the condition of intermittent hypoxia (5% O_2_), cells were incubated in a humidified incubator (Heracell Vios 160i Co2-Incubator, Thermo scientific, Waltham, MA, USA) at 37 °C for 1 h. Further, for chemically induced hypoxia, THP-1 cells were incubated with CoCl_2_ (100µM; Sigma Aldrich Chemie GmbH, Hamburg, Germany) for 1 h, respectively. Native 1,8-Cineol extract (0.6 mg/µl 1,8-Cineol) was stored at 4 °C, while stock solution was prepared by solving native extract in ethanol (100 mg/ml) followed by a final dilution with DMEM high glucose (Biochrom, Berlin, Germany; 1 mg/ml 1,8-Cineol).

### Total RNA extraction and quantitative real-time PCR

Total RNA from THP-1 monocytes was extracted using the innuPREP RNA Mini Kit 2.0 (IST Innuscreen GmbH, Berlin, Germany) according to the manufacturer’s protocol. cDNA of total RNA (100–300 ng) was synthesized with the M-MuLV reverse transcriptase (New England Biolabs, Frankfurt, Germany) and random hexamer primers (ThermoFisher Scientific) according to the instructions of the manufacturer. The sequences of forward and backward primers are listed in Table 1.

Primers were synthesized by Invitrogen (USA). Quantitative RT-PCR was performed in the Eco48 qPCR System (PCRmax Limited Beacon Road, Staffordshire, United Kingdom) using 1 µL cDNA and the SensiMix SYBR Kit (Bioline, Luckenwalde, Germany) in a total volume of 12.5 µL per assay. The cutoff point (Ct) was defined as the value at which the fluorescent signal increased above the background threshold. Gene-specific mRNA expression of hypoxia inducible Glut1 was normalized to mRNA expression of ribosomal protein L28 (RPL28). Relative expression values were calculated using the 2^∆∆ct^-method and are presented as the fold induction to L28 compared with THP-1 monocytes cultured in normoxia.

**Table 1:** Sequences of forward and backward primers.

Primers	Sequences
GLUT1	
Forward	5‘- GGCCTTTTCGTTAACCGCTT-3‘
Reverse	5‘- AGCATCTCAAAGGACTTGCCC − 3‘
L28	
Forward	5‘- ATGGTCGTGCGGAACTGCT − 3‘
Reverse	5‘- TTGTAGCGGAAGGAATTGCG − 3‘

### Staining of THP-1 cells and FACS analysis

THP-1 cells were stained with the following antibodies (diluted 1:50): CX3CR1-BV421 (Cat: 341620), CD11a-PE-Cy7 (Cat: 301220), CD11b-PerCP (Cat: 101230), CD11c-BV421 (Cat: 371512), and PD-L1-APC (Cat: 304308) (all from BioLegend, San Diego, USA). After 20 min of staining in the dark, 500 µl RBC Lysis Buffer (BioLegend) were added to the samples and incubated for another 20 min. Subsequently, suspension was centrifuged at 400 x g for 5 min at 4 °C and supernatant was discarded. Cell pellet was resuspended in 100 µl fresh PBS and used for FACS analysis. Flow cytometry was performed with a MACSQuant 10 flow cytometer (Miltenyi Biotec, Bergisch-Gladbach, Germany) and data were analyzed using the FlowJo software version 10.0 (FlowJo, LLC, Ashland, USA).

### Cytokine analysis

Cell culture supernatants were collected and instantly frozen with liquid nitrogen and preserved at -80 °C. Analyses of cytokines and chemokines were performed using membrane based Proteome Profiler^™^ Human XL cytokine arrays (R&D Systems, Minneapolis, MN, USA) as recommended by the supplier. Expression was visualized using an enhanced chemiluminescence detection kit (R&D Systems, Minneapolis, United States). Semiquantitative analysis was performed by measuring the density of the dots using an iBright CL 1000 biomolecular imager (Invitrogen, Carlsbad, CA, USA). Plasma concentrations of growth hormone (GH) were assessed from citrate-plasma samples and were determined by enzyme-linked immunosorbent assays (ELISA) according to manufacturer’s protocols (R&D Systems, Minneapolis, MN, USA).

### Statistical analyses

Statistical analyses were performed with GraphPad Prism Version 7.0 f. The mean and standard error (SEM) are presented. The differences between groups were determined after testing for Gaussian distribution (normality tests), and applying parametric (student`s t-Test), or non-parametric 1-way ANOVA with Bonferroni post hoc test. The correlation between parameters was calculated using multivariate regression with the Pearson correlation coefficient. *p* < 0.05 (*), *p* < 0.01 (**), and *p* < 0.001 (***). Additional statistical details such as sample size are given in the respective figure legends, when appropriate.

## Data Availability

Data is provided within the manuscript.
